# Kelvin probe force microscopy of the nanoscale electrical surface potential barrier of metal/semiconductor interfaces in ambient atmosphere

**DOI:** 10.3762/bjnano.10.138

**Published:** 2019-07-15

**Authors:** Petr Knotek, Tomáš Plecháček, Jan Smolík, Petr Kutálek, Filip Dvořák, Milan Vlček, Jiří Navrátil, Čestmír Drašar

**Affiliations:** 1 University of Pardubice, Faculty of Chemical Technology, Department of General and Inorganic Chemistry, Studentská 573, 532 10 Pardubice, Czech Republic; 2 University of Pardubice, Faculty of Chemical Technology, Joint Laboratory of Solid State Chemistry, Studentská 84, 532 10 Pardubice, Czech Republic; 3 University of Pardubice, Faculty of Chemical Technology, Center of Materials and Nanotechnologies, Studentská 95, 532 10, Pardubice, Czech Republic; 4 Institute of Macromolecular Chemistry of the Academy of Sciences of the Czech Republic v.v.i., Heyrovsky sq. 2, 12006 Prague, Czech Republic; 5 University of Pardubice, Faculty of Chemical Technology, Institute of Applied Physics and Mathematics, Studentská 95, 532 10, Pardubice, Czech Republic

**Keywords:** Kelvin probe atomic force microscope, nanoinclusion, Schottky barrier, thermoelectric materials, work function

## Abstract

This study deals with the preparation and characterization of metallic nanoinclusions on the surface of semiconducting Bi_2_Se_3_ that could be used for an enhancement of the efficiency of thermoelectric materials. We used Au forming a 1D alloy through diffusion (point nanoinclusion) and Mo forming thermodynamically stable layered MoSe_2_ nanosheets through the reaction with the Bi_2_Se_3_. The Schottky barrier formed by the 1D and 2D nanoinclusions was characterized by means of atomic force microscopy (AFM). We used Kelvin probe force microscopy (KPFM) in ambient atmosphere at the nanoscale and compared the results to those of ultraviolet photoelectron spectroscopy (UPS) in UHV at the macroscale. The existence of the Schottky barrier was demonstrated at +120 meV for the Mo layer and −80 meV for the Au layer reflecting the formation of MoSe_2_ and Au/Bi_2_Se_3_ alloy, respectively. The results of both methods (KPFM and UPS) were in good agreement. We revealed that long-time exposure (tens of seconds) to the electrical field leads to deep oxidation and the formation of perturbations greater than 1 µm in height, which hinder the *I*–*V* measurements.

## Introduction

Increasing energy demand and the negative effects of current energy technologies on the environment lead to increased interest in alternative energy technologies. Thermoelectric (TE) devices, utilizing TE phenomena, i.e., the Seebeck and the Peltier effect, can be considered solid-state heat engines. The former enables direct waste heat conversion into electrical energy very elegantly (no moving parts, long-term stability, maintenance-free, silent). Unfortunately, one of the limiting factors of this technology is still poor conversion efficiency. The conversion efficiency of the material could be expressed in terms of figure-of-merit, ZT, defined as dimensionless quantity ZT = *S*^2^·σ·*T*/κ , where *S* is the thermopower (Seebeck coefficient), σ is the electrical conductivity, *T* is the absolute temperature and κ is the thermal conductivity. There were many concepts for enhancement of ZT values, e.g., advanced TE bulk compounds of more complex crystal structures [1], low-dimensional material systems [2] or nanostructured advanced bulk materials [3]. Recently the concept of multi-phase nanocomposites (NCs), i.e., an incorporation of second-phase nanoinclusions/nanoparticals (NIs/NPs) into the bulk semiconducting matrices has been proposed [4–6] and supported in theoretical works [7–9]. Such NIs or NPs are able to enhance ZT via a reduction of the thermal conductivity by phonon scattering [10–14], by modulated carrier doping or by the carrier energy filtering effect [15–17]. The potential energy barrier connected with a metallic NP (Schottky barrier) or a multi-phase interface could scatter low-energy electrons more effectively than high-energy electrons [18]. This, in turn, results in an enhancement of the Seebeck coefficient with virtually no harm to other transport parameters.

The characterization of NIs or NPs in TE materials is realized most frequently by the different modes of atomic force microscopy (AFM): i) by comparing the conductivity/resistivity (CAFM) or *I*–*V* curves measurement in the direct-contact of the conductive tip and the material [19–21]; ii) by mapping of the different surface contact potential values by Kelvin probe force microscopy (KPFM) in the semicontact mode [19,22–25], or iii) by measuring the differences in thermal conductivity by scanning thermal microscopy (SThM) [19–20,26]. Shape, size, homogeneity of distribution and chemical composition as the basic characteristics are studied by using electron microscopy (SEM+TEM), although there is a high probability that the NPs diffuse into the TE matrix due to the e-beam interaction. Hence, these characterizations are also performed by using scanning tunneling microscopy (STM) [27–28] or by using AFM in the semicontact mode. The latter enables a describtion not only of the topography (size and shape) but also a detection of the changes in density, stiffness and adhesion of NPs [20–21,24,29–30].

In the present study we demonstrate that the Schottky barrier (surface contact potential) value and the polarity can be controlled by the barrier-forming metal NPs (Au, Mo) and can reflect their different chemical behavior with the Bi_2_Se_3_ matrix. These metals were selected due to the different interaction with the matrix, as Au can diffuse to the Bi_2_Se_3_ forming Au/Bi_2_Se_3_ alloy and NIs in the shape of a semispherical defect, whereas Mo reacts with the matrix and forms thermodynamically more stable layered MoSe_2_ in the shape of nanosheets.

## Experimental

Single crystalline Bi_2_Se_3_ samples were grown by heating stoichiometric mixtures of the pure elements obtained from Sigma-Aldrich, i.e., 5N Bi and 5N Se. The crystal growth was performed by cooling in a horizontal furnace from 1073 K to 823 K at a rate of 6 K per hour. The crystals were then annealed at 823 K for 350 h and quenched in air. This free-melt crystallization (FMC) procedure produces single crystals of 10–20 mm in length, 3–6 mm in width and up to 3 mm in thickness [31]. The surface of freshly cleaved layered Bi_2_Se_3_ single crystals was used as a substrate. The samples for the AFM measurement, Au nanoparticles (NPs) and thin films of gold or molybdenum, were prepared via DC sputtering in a SEM Coating System (Bio-Rad) in Ar atmosphere (*p* ≈ 20 Pa, *I* = 18 mA, *U* = 1.4 kV) from pure metal sheets (Mo 4N, Au 4N). For preparing separated Au nanoparticles under the same sputtering conditions, however, a stainless steel mask (system of 100 × 500 μm^2^ holes, 200 holes/cm^2^) between the substrate and target had to be used to decrease the plasma intensity.

The topography, phase shift image, Kelvin probe force microscopy and *I*–*V* characteristics were measured by the AFM SolverPro M, Nt-MDT (Russia) with a resolution of 512 × 512 pixels. The HA_NC tips (resonant frequency 140 kHz, force constant 3.5 N/m) were used for measuring the metal layer thickness by the scratch method and phase contrast [30,32], while conductive NSG01/TiN tips (150 kHz, 5.1 N/m) were used for Kelvin probe force measurements and contact CSG-10/Pt tips (22 kHz, 0.11 N/m) for recording *I*–*V* characteristics [33–34] for 10 replicas from an identical point without delay; see the corresponding references above for other experimental details. The set-point was stabilized in the range of 40–50% and the scanning frequency of 0.5 Hz was used, if not mentioned otherwise. A separation of the topographical signal and the *V*_CPD_ (the contact potential difference) measurement was achieved by the modulation of the *V*_AC_ at a frequency higher than the bandwidth of the topography feedback system. The topography was measured by the oscillation at the first resonance frequency of the AFM tip, and *V*_CPD_ was measured by the amplitude of the oscillation at the second resonance frequency of the AFM tip [35–36]. It also has to be noted that all measurements were carried out at room temperature.

Photodiffusion of the metal into Bi_2_Se_3_ was enabled by means of a Ronchi ruling (non-transparent Cr lines on the SiO_2_ substrate) with a density of 2000 lines per millimeter (period 500 nm) as a photomask. The material was illuminated with a UV light source LC08 (Hamamatsu) emitting at wavelengths of 310 and 360 nm; total intensity 0.8 W/cm^2^ for 60 s [37–38]. The e-beam diffusion was realized by a SEM JEOL JSM 5500-LV in an of area 8 × 10 µm^2^ with an acceleration voltage of 20 kV (which results in a penetration depth of the electrons of 1 µm into Bi_2_Se_3_) for a period of maximally 360 s.

The work function (WF) was determined by utraviolet photoelectron spectroscopy (UPS) using a helium gas discharge source with He I radiation (*h*ν = 21.22 eV). All UPS measurements were performed in an UHV apparatus (ESCA 2SR, Scienta-Omicron) with a base pressure below 1 × 10^−9^ mbar. The spectra were calibrated using the Fermi edge of sputter-cleaned Au as reference. During the measurements, a bias of −10 V was applied to the sample in order to cut off secondary electrons generated in the analyzer. The work function of the sample was calculated as WF = *h*ν *E*_cut-off_, where *E*_cut-off_ was determined from the intersection of the linear extrapolation of the secondary-electron cut-off (SECO) with the background. All samples were sputtered with argon ions using a scanning focused ion beam source in order to remove surface contaminants. A monoatomic argon ion source was utilized with energy of 2 keV, ion current 10 µA, raster area 1 × 1 mm^2^ and sputtering time 30 s.

## Results and Discussion

### Separated metal nanoparticles on the substrate

In TE materials the NIs applicable for an increase of their profitability/efficiency should be present as 1D materials or 2D layers. The aim of the characterization of the NIs is the proof of their presence, the description of their shape and of their electrical behavior in contrast to the unaffected matrix material. We used Bi_2_Se_3_ as a standard material because of its well-defined layered structure with sub-nanometer roughness similar to mica or highly oriented pyrolytic graphite (HOPG). The electrical conductivity and mechanical stiffness of Bi_2_Se_3_ allow for the measurement with high-intensity electrical fields (10 V/30 nm) without damaging the topmost layer.

**Figure 1:**
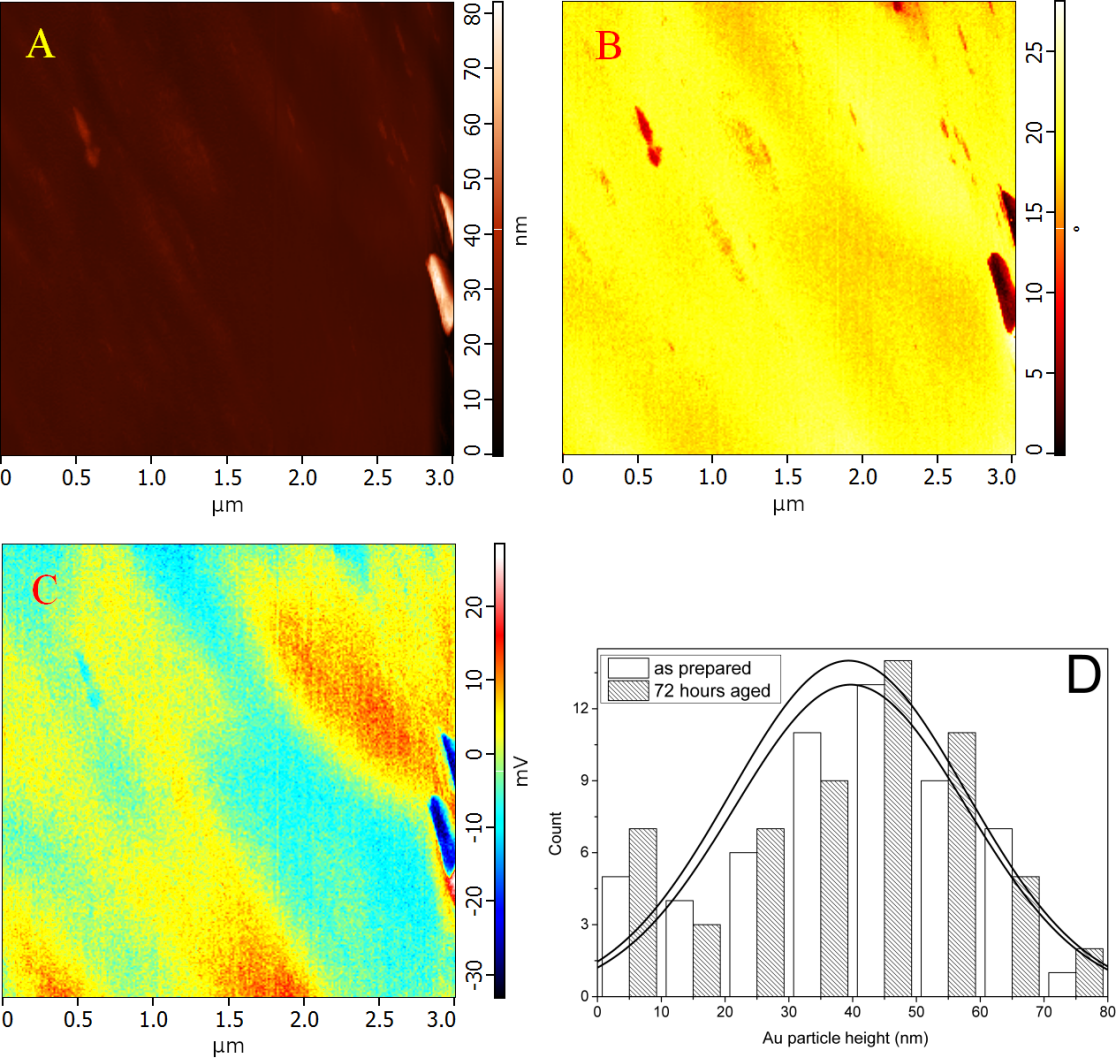
(A) Topographical and (B) phase-shift image, and (C) surface contact potential map of Au nanoparticles on Bi_2_Se_3_ (as prepared); (D) histogram of the Au NP height distribution for the as-prepared sample and after 72 h.

The Au particles were dc-sputtered onto the Bi_2_Se_3_ substrate and the surface was immediately measured by means of AFM. The particles exhibited a height ranging from 5 to 70 nm. The height distribution (with the maximal population at ca. 40 nm) and the FWHM did not change within 72 h after preparation (Figure 1A and Figure 1D). The stability of the histograms over 3 days led us to believe that during this time interval there is only negligible diffusion of the Au NPs into the Bi_2_Se_3_ substrate. The material of the particles was mechanically different as observed on the phase shift image (Figure 1B) due to local changes in adhesion, density, and stiffness [30]. This fact demonstrates the difference between the NPs and the substrate, regardless of any artifacts of the sample preparation/measurement.

**Figure 2:**
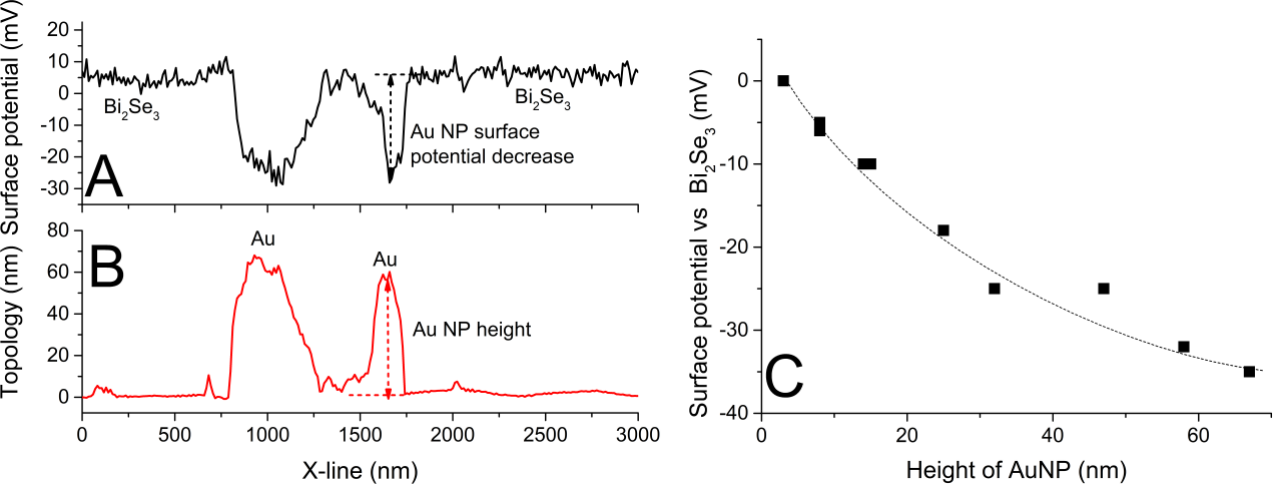
(A, B) Determination of the surface contact potential decrease and the height of the Au NPs; (C) surface contact potential as a function of the Au NP height.

KPFM is a double-pass measurement technique, where the lift height between the first (topographical) and second (electrical) pass is an important parameter for the spatial resolution [36,39–40]. The tested lift heights were 10, 30 and 50 nm, and the optimal value was found to be 30 nm (Figure 1C). The KPFM image has a good contrast for a lift height of 30 nm and particles with a height less than 10 nm were detected in the map of the surface contact potential. A higher lift height led to a vanishing of the contrast and a lower value resulted in a decrease in reproducibility (data not shown). The results are in a good agreement with the KPFM contrast of nanodiamonds on a Si substrate, where the contrast even changed for the lower lift height (see Figure 10.2 in [41]). The atypical conductive TiN coating of the tip (instead of Pt, Au Pt/Ir or Pt/Cr tips [33,41–43]) was used because of the higher mechanical stability of the tips and in order to eliminate diffusion and other chemical interactions of the tip coating with the samples. The measured difference of the surface contact potential (Figure 1C) between Au particles and Bi_2_Se_3_ was not identical for all particles depended on the height of the particles. The height, i.e., the vertical distance between the top of the NP and the substrate and the contrast of the surface contact potential of Au particles (Figure 2A, B) were measured within the same line profile. Au NPs of greater height showed lower values of the surface contact potential according to the non-linear curve limited to a Δ surface contact potential between −50 and −60 mV. This observation can be attributed to quantum size effects [36,44], which are accompanied by a change in the charge transfer from substrate to NPs [45–46].

### Metal layers on Bi_2_Se_3_

The dependence of the surface contact potential on the size of the separated Au nanoparticles theoretically allows for an optimization of the efficiency of TE materials. Our efforts to prepare separated Mo NPs on the Bi_2_Se_3_ surface was not successful. Because Mo has significantly higher melting/boiling points (bulk Mo 2623/4639 °C, bulk Au 1064/2856 °C [47]) dc sputtering yielded homogeneous layers of Mo instead of separated NPs. In addition, the preparation from colloidal metallic NPs failed as the solvents or stabilizing agents as polyphosphates or vinylpyrrolidones [48] interacted with the uppermost Bi_2_Se_3_ layer, or residua of the organic solvents that were not evaporated under the vacuum changed the electrical behavior of the nanocomposite.

**Figure 3:**
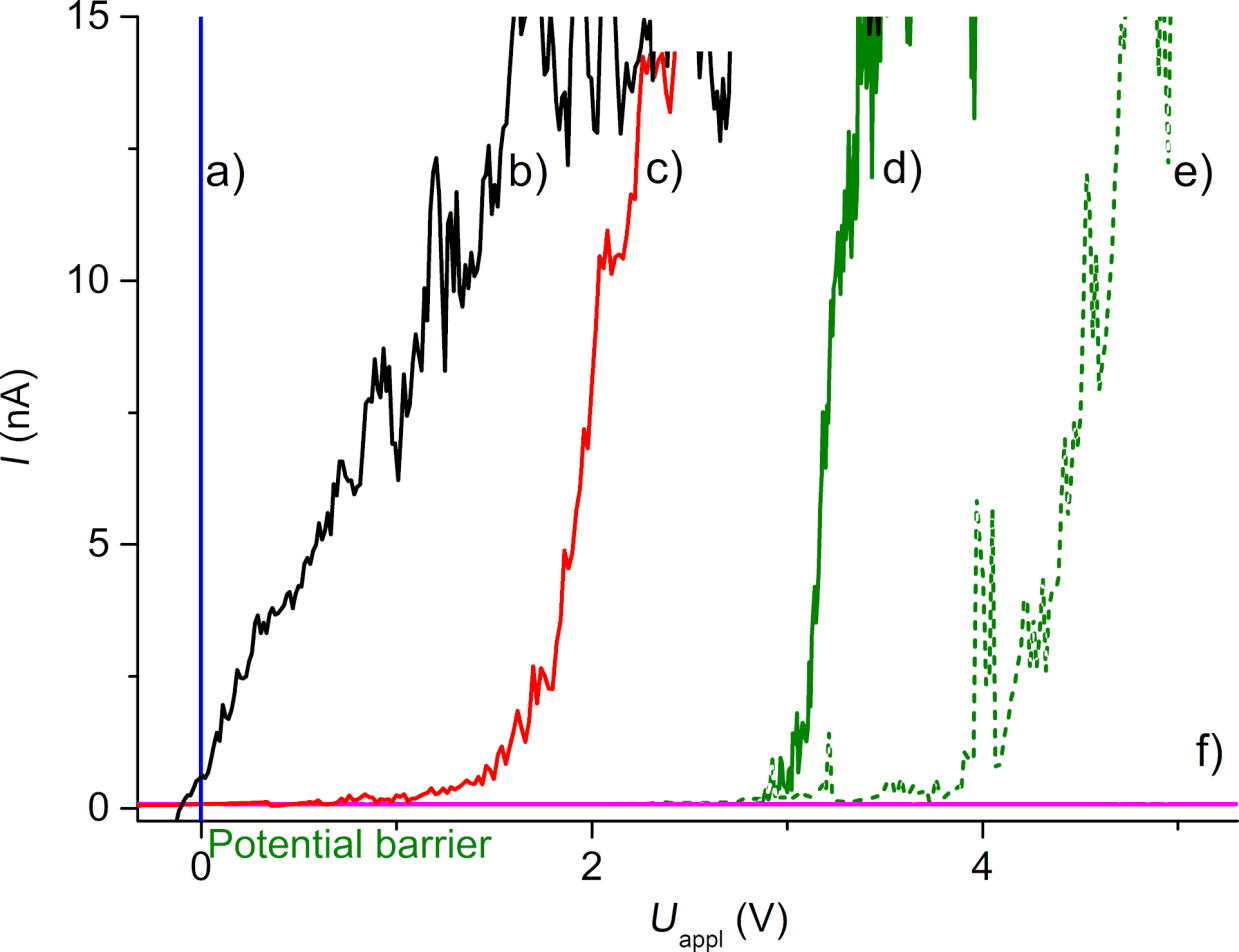
Single-point *I*–*V* curves performed during the mechanical contact with the Pt-coated conductive tip on a) bulk Au; b) 107 nm Au layer (both contacts on the same layer); c) 53 nm Au layer; d) and e) 19 nm Au layer before and after e-beam diffusion; f) cleaved Bi_2_Se_3_. Curves c–f) were measured with the ohmic contact on the 107 nm Au layer (see Figure S1, Supporting Information File 1).

An alternative way to study Schottky barriers at the nanoscale is to prepare a metallic layer on the Bi_2_Se_3_ surface and characterize such a nanocomposite. *I*–*V* spectroscopy is typically used for the determination of Schottky barriers [25,49–50]. The setup was tested on bulk Au and on a 107 nm Au layer on the Bi_2_Se_3_ (for both cases the contact electrode and conductive tip were located on the same material). We observed a higher conductivity of the bulk material (quantum size effect) (Figure 3, compare curves a) and b)) and a small photodiode effect of Au/Bi_2_Se_3_ (−100 mV) due to the AFM illumination (Figure 3, see the intersection of curve b with the *x*-axis). We also measured Au layers with a thickness of 53 nm and 19 nm (before and after e-beam forced diffusion), and bare Bi_2_Se_3_ (contact placed on the 107 nm Au layer), see Figure S1, Supporting Information File 1. The curves for each Au thickness at the same area with the best reproducibility are shown in Figure 3. The potential cut-in barrier strongly depends on the Au thickness with values of 1.5 V for the 53 nm Au layer and 3.0 V for 19 nm Au (see Figure 3; curves c, d)). No potential barrier was measurable on Bi_2_Se_3_ up to 6 V.

**Figure 4:**
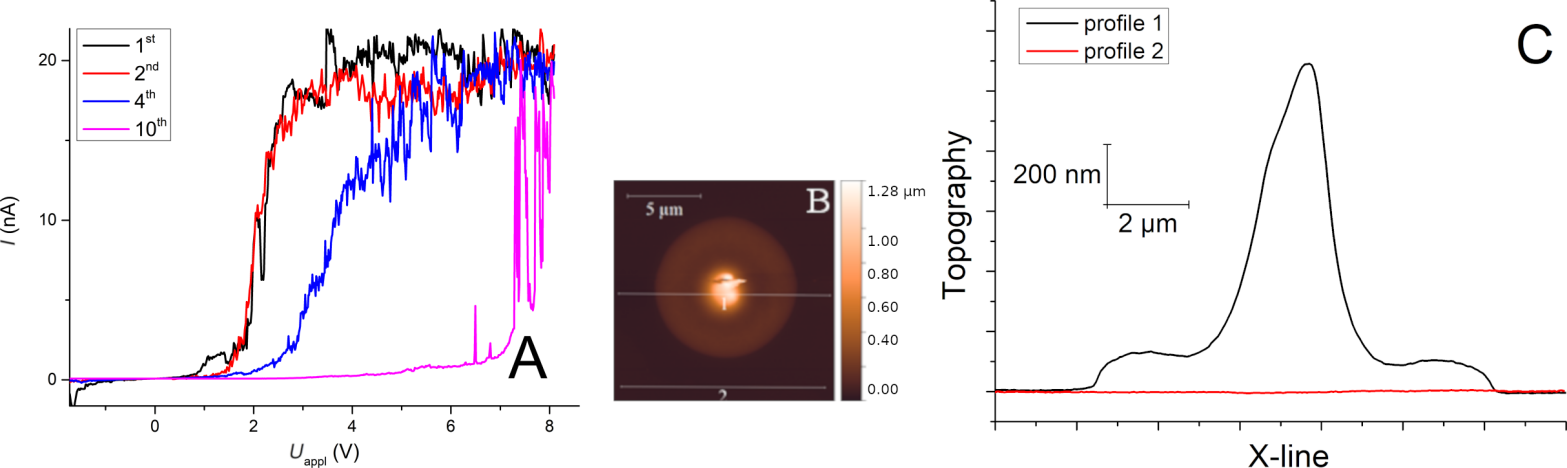
(A) Change of the *I*–*V* characteristics for ten repetitions of a single-point and (B) induced changes in the topography and (C) height profiles across a perturbation and a non-affected area (horizontal lines in (B)).

There is a systematic increase in the potential cut-in barrier for 53 nm Au/Bi_2_Se_3_ upon cycling (Figure 4A). The cut-in barrier was less than 1 V during the first measurement, and above 6 V for the tenth measurement at the same place. The topography also changed significantly after 10 cycles of *I*–*V* measurements. Newly formed perturbations were observed with a height of over 1 µm and a FWHM of 2 µm (Figure 4B). The changes in the electrical behavior as well as the growth of the perturbations can be explained by the growth of an isolating oxide layer by anodic oxidation. This has also been observed for Si [51–52], Ti, Ni or Al [53–55], carbon [56] and organic–inorganic compounds [57]. We suggest, in our case, that OH^−^ or O^−^/O_2_^−^ ions diffuse through our polycrystalline Au layer with many defects (grain boundaries, dislocations) forced by the strong electric field (8 V/53 nm of Au, i.e., ca. 1.5 × 10^8^ V/m) [35–36]. At the Au–Bi_2_Se_3_ interface the oxidation of Bi_2_Se_3_ can occur accompanied by the formation of non-conductive bismuth oxides (optical *E*_g_ = 3.31 eV for a 60 nm BiO*_x_* film [58]). Typically, the thickness of surface oxide layers is much lower (of the order of nanometers for SiO_2_ on Si [51–52]). In our case it is increased by the electric field and good oxygen mobility in BiO*_x_* due to the formation of charged Bi vacancies [59–60].

This assumption is in good agreement with the observed increase of the cut-in potential barrier upon thinning of the Au layer (see Figure 3) as a reminiscence of the diffusion limitation of oxygen/hydroxy anions due to the electric field through the polycrystalline Au layer. Bare Bi_2_Se_3_ had no diffusion limits and the *I*–*V* curves are not measurable as electrons immediately form isolating BiO*_x_* on the surface. The abovementioned facts, tentatively summarized as *I*–*V* characteristics, are strongly affected by the oxidation of Bi_2_Se_3_. The perturbation with a height of more than 1 µm could be easily formed by the oxidation of a highly O-mobile material, e.g., BiO*_x_*. This limits the usage of *I*–*V* measurements in the static electrical contact of the Schottky barrier in ambient atmosphere to stable materials.

KPFM is an alternative way for mapping the changes in the surface contact potential [19–20,26] and reduces force and time of the tip–sample interaction, which in turn reduces the oxidation of the material. The NIs were embedded by local Au diffusion or the reaction of the Mo layer with the Bi_2_Se_3_ substrate. To enhance the diffusion/reaction rate, we used e-beam irradiation. An area of the 8 × 10 µm^2^ was locally irradiated by an electron beam for different periods of time (75 s; 180 s and 360 s). This resulted in a nanocomposite material consisting of the metal layer locally reacted with Bi_2_Se_3_ support and the original metal layer on the support surface. We were able to compare metallic materials with different thicknesses and determine the highest contrast in the surface contact potential reducing the problem of the diffused interface.

**Figure 5:**
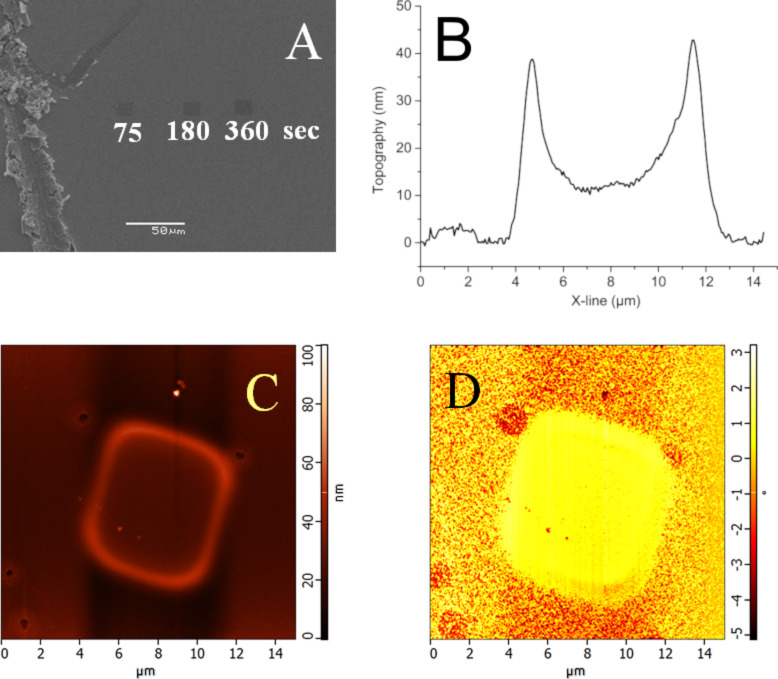
(A) SEM image of the Au layer (33 nm in thickness) on Bi_2_Se_3_ irradiated by the electron beam for different periods of time; (B) topographical profile across the irradiated area taken from (C); (C) topographical AFM and (D) and phase-contrast AFM image of the e^-^beam-irradiated (360 s) Au layer (33 nm in thickness) on Bi_2_Se_3_.

**Figure 6:**
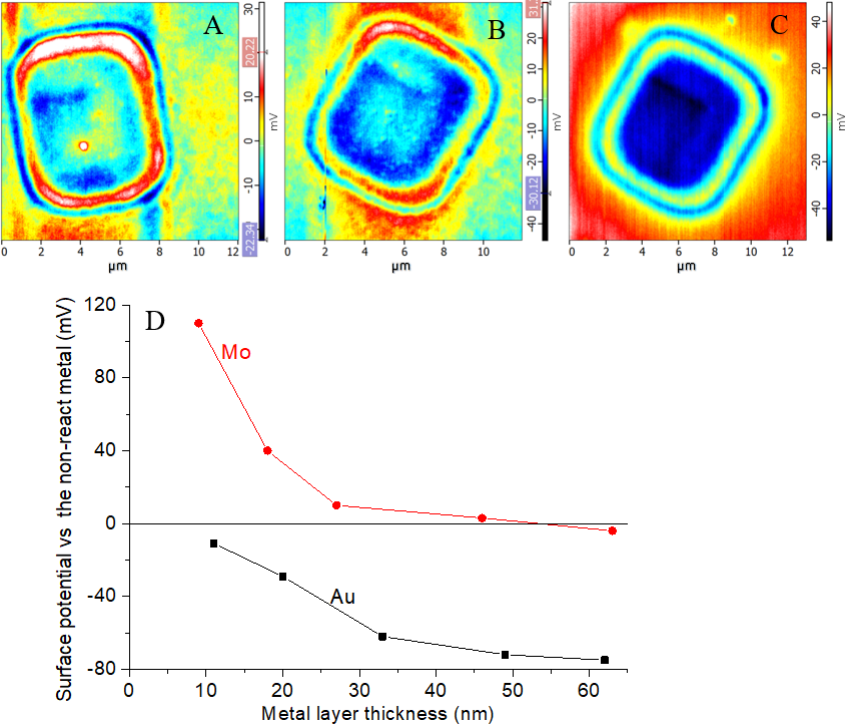
Maps of the surface contact potential measured by means of KPFM for the e-beam formed NIs on the Au layer/Bi_2_Se_3_ for different values of Au layer thickness: (A) 12 nm, (B) 20 nm (B), and (C) 33 nm; (D) surface potential contrast between irradiated and non-irradiated parts of the metal layers as a function of the layer thickness.

The e-beam-irradiated area exhibits a different SEM signal due to the changes of the chemical composition (Au diffusing into Bi_2_Se_3_) and changes in the topography (Figure 5). The same NIs, detected by the AFM (Figure 5), exhibited the topographical features of a few tens of nanometers (10 nm in the center and almost 40 nm at the edges) and changes in the phase contrast, both due to the density decrease of the Au alloy upon reaction with the substrate. All samples were irradiated for a period of 360 s. KPFM demonstrated strong variations of the surface contact potential of NIs (measured in the center of the irradiated area) with respect to the non-irradiated area of the Au layer (Figure 6). It should be noted, that the edges of the irradiated area are topographically elevated and exhibit a decreased surface potential because of the higher electron irradiation dose at the turning points of the e-beam. A higher dose led to a higher local temperature, which implies an increased formation of Au alloy and rims. The comparable so-called “Marangoni effect” was observed in laser-irradiated polymers as described by Lyutakov and co-workers [61].

The NIs formed on the thinnest Au (12 nm) exhibited a surface potential signal slightly above the detection limit of KPFM (Figure 6). With increasing thickness of the Au layer there is a higher contrast (Figure 6). The maximum value of the surface potential difference is between −80 and −90 mV and is connected with the diffusion of Au into Bi_2_Se_3_. Although the dependence and the values are alike for 1D particles (Figure 2) and for layers (Figure 6), it is difficult to compare them due to the different “substrate” (Bi_2_Se_3_ for 1D particles and non-diffused Au for the layers).

**Figure 7:**
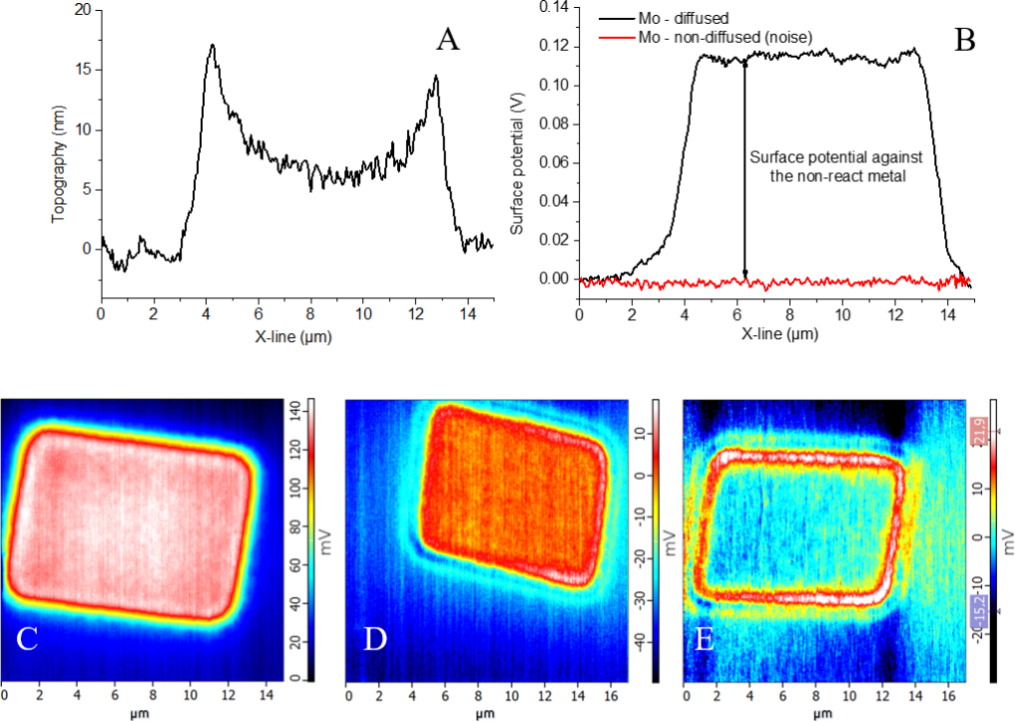
(A) Topographical and (B) surface contact potential profiles of the 9 nm irradiated Mo layer; maps of the surface contact potential measured by the KPFM for the e-beam formed NIs on the Mo layer/Bi_2_Se_3_ for Mo thickness values of (C) 9 nm, (D) 17 nm, and (E) 63 nm.

The Mo layers on Bi_2_Se_3_ reacted to the e-beam irradiation in a different way. The irradiated part of the Mo layer expanded less strongly than the Au layer (5 nm at the center, 20 nm at the edge, see Figure 7 profiles) and the contrast in the surface contact potential increased. The most intensive contrast (109 mV) was detected for NIs in the 9 nm thick Mo layer, whereas almost negligible surface contact potential difference (−4 mV) was detected for the 63 nm thick Mo layer.

**Figure 8:**
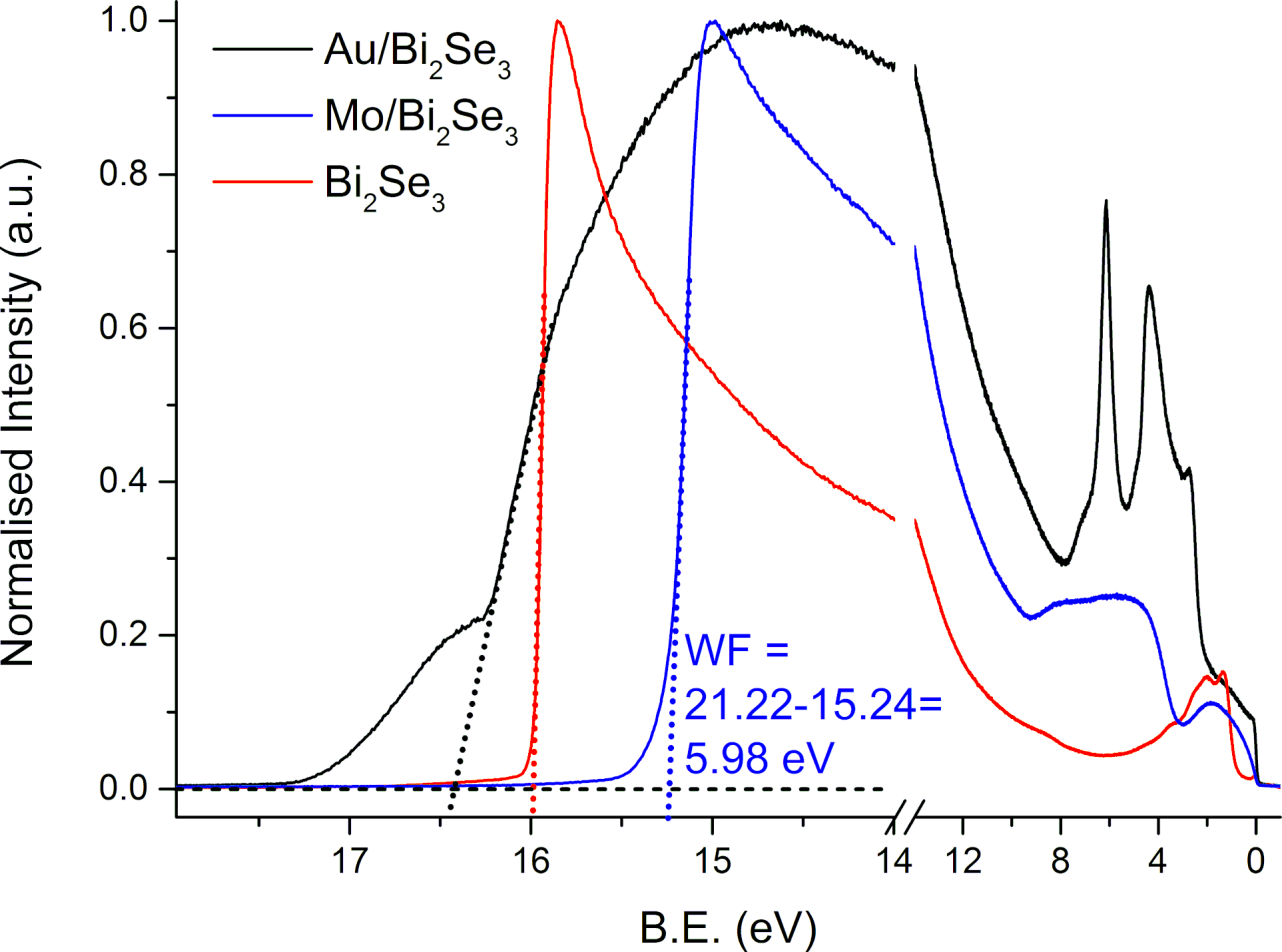
UPS data for Bi_2_Se_3_; Mo or Au layers on the top of the Bi_2_Se_3_ cleaned by the Ar^+^ sputtering excited by the He I (*h*ν = 21.22 eV).

The results of the surface contact potential mapping at the nanoscale were correlated to the macroscopic work functions (WF) of freshly cleaved Bi_2_Se_3_ and Au or Mo layers (both 30 nm in thickness). Work functions were measured by surface-sensitive ultraviolet photoelectron spectroscopy (UPS) (Figure 8) employing He I irradiation (*h*ν = 21.22 eV) with an information depth up to 3 nm. The work function was determined from UPS spectra as an intersection between the background and the linear extrapolation of the secondary-electron cut-off. We obtained WF_Bi2Se3_ = 5.22 eV, WF_Au/Bi2Se3_ = 4.79 eV, WF_Mo/Bi2Se3_ = 5.98 eV for Ar^+^-sputtered samples. The WF increased by 760 meV with the Mo layer, whereas the WF decreased by −430 meV after sputtering the Au layer onto Bi_2_Se_3_.

We can sum up above results as follows:

The Au layer on the Bi_2_Se_3_ surface exhibited lower work function (WF) values than the freshly cleaved Bi_2_Se_3_ surface, measured by means of the UPS (4.79 eV vs 5.22 eV). This is in good agreement with the decrease of the surface contact potential of the separated Au particles on Bi_2_Se_3_ detected by the KPFM (limited to −40 mV, see Figure 2C).

The local irradiation of the Au layer on Bi_2_Se_3_ (Ar^+^ ions before UPS or SEM electrons during formation of NIs for KPFM measurements) led to a reaction with a substrate, most likely the incorporation of interstitial Au atoms into the Bi_2_Se_3_ matrix and Au alloy/compound formation. This material displayed lower density (topographical expansion, Figure 5) and the creation of the defect states (tentatively connected to the shoulder of the UPS edge, see Figure 8, black curve near 17 eV as well as a decrease in the surface contact potential of the Au NIs, see Figure 6).

In the case of Mo the situation is more complex. We were not able to prepare separated Mo 1D particles due to the higher melting temperature of Mo compared to the Au. The thinnest Mo layer led to the highest contrast in the surface contact potential of the NIs (see Figure 7) due to the formation of a thermodynamically more stable MoSe_2_ layer. The increase in Mo layer thickness led to the presence of the metallic Mo, which is not stable in air and is oxidized within 1 h at RT [62]. The presence of the MoO*_x_* layer is reflected by the high value of the WF (5.98 eV in this work, Mo 4.3 eV and MoSe_2_ 5.65 eV as reported in [63]), which fits better to MoO*_x_* layer after the thermal evaporation (6.8 eV) and after 1 h oxidation at air at RT (5.6 eV). Partly oxygen-depleted MoO*_x_* exhibited a WF of 6.36 eV [62]. In our case, non-reacted Mo on the stable MoSe_2_ remains reactive (layered MoSe_2_ forms a barrier for further Mo diffusion) and Mo reacts with oxygen to produce MoO*_x_*. The UPS information is obtained from the topmost 2–3 nm, i.e., from the oxidized MoO*_x_* layer (30 nm of Mo was sputtered on the Bi_2_Se_3_ for UPS measurements). The 9 nm Mo layer after SEM electron irradiation led to the formation of MoSe_2_ (through the reduction of MoO*_x_* in vacuum to Mo and subsequent reaction with Bi_2_Se_3_) and these NIs revealed the highest contrast in contact potential (Figure 7). When the thickness of the Mo layer (17 and 63 nm, see Figure 7) is increased, not all Mo reacts to MoSe_2_ and there is a cover layer of MoO*_x_*. If the MoO*_x_* layer is irradiated by electrons under the same conditions as the thinnest Mo layer, there will be a partial oxygen depletion as well [62]. However, the long distance from the Bi_2_Se_3_ substrate hinders a reaction (the sample exhibits a sandwich structure of reactive Bi_2_Se_3_/isolating MoSe_2_/isolating Mo and MoO*_x_*/reactive oxygen-depleted MoO*_x_*). This is further reflected in a low contrast in KPFM measurements, as only non-irradiated and re-oxidized (due to ambient atmosphere) MoO*_x_* layers are measured. Even if MoSe_2_ is formed at the MoO*_x_*/Bi_2_Se_3_ interface, its presence is hidden for both UPS (low information depth) and KPFM (electrons prefer a more conductive path in the Mo/MoO*_x_* layer).

**Figure 9:**
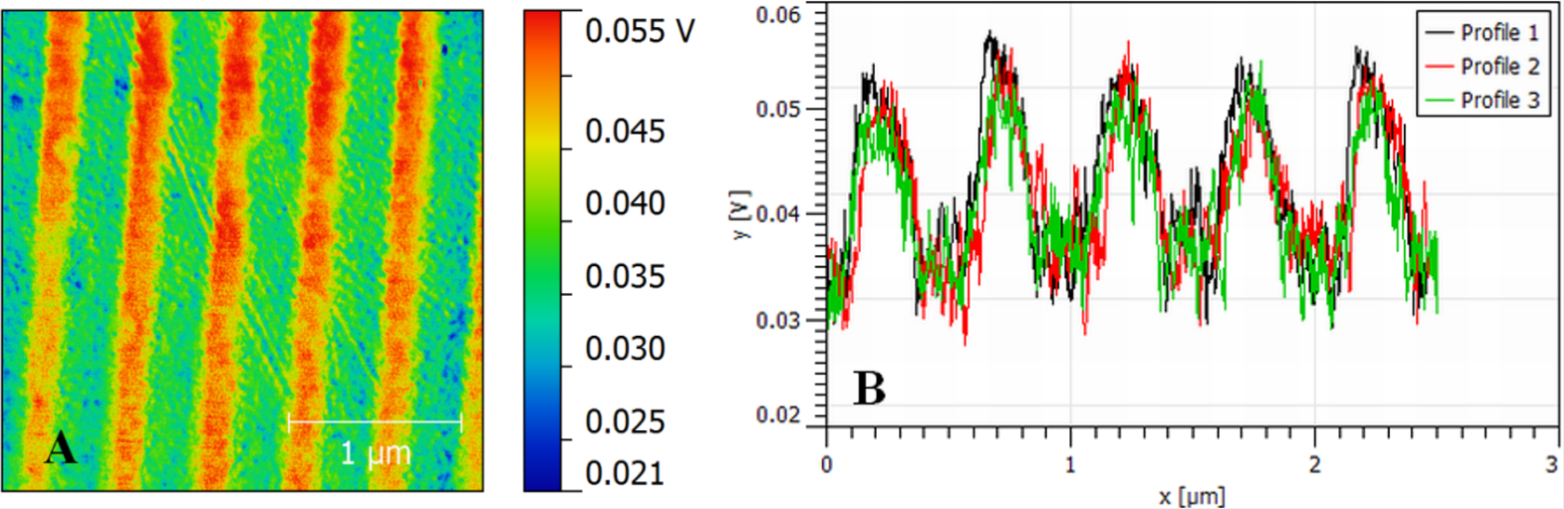
(A) Changes of the surface potential in the pattern after UV photodiffusion of Au (20 nm) into Bi_2_Se_3_ and (B) three line profiles.

The applicability of the partial diffusion of the Au layers was tested by the photodiffusion of the 20 nm Au layer into Bi_2_Se_3_ by UV source through a Ronchi ruling [64–65]. The illuminated Au–Bi_2_Se_3_ exhibited a periodical pattern of the surface potential with an amplitude of 20 mV (Figure 9). This value is slightly lower in contrast to the NIs obtained by e-beam irradiation of the 20 nm Au layer (decrease 29 mV), and could be connected with the partial lateral diffusion of the heat or Au atoms/ions. In this way, the possibility to prepare a material with patterned surface contact potential at the macroscopic scale was demonstrated.

## Conclusion

We demonstrated local changes of the work function/surface contact potential as a consequence of metal nanoinclusions on the surface of Bi_2_Se_3_. These Schottky barriers can be realized in the form of separated metal nanoparticles through dc sputtering, or through the local reaction/diffusion of a metallic layer irradiated with electrons or UV photons. The value of the work function barrier is measurable by KPFM at the nanoscale even in ambient atmosphere and, most importantly, it strongly depends on the size of the metal particles. The results are in good agreement with the macroscopic UPS results (1 mm^2^) obtained in UHV. The investigation of the Schottky barrier in ambient atmosphere is limited to noble metals only, as the Mo nanolayer reacts with oxygen forming MoO*_x_* at RT within a few hours. We therefore conclude that KPFM has the following limitations: i) Only oxidization-resistant metals/semiconductor nanoinclusions can be used. Transition metals react with air and, thus, vacuum equipment is required for the preparation, transport and measurement. ii) The conductive AFM tip has to be coated with a mechanically hard and non-reacting layer as TiN. Typical Au or Pt coatings need to be avoided because the coating might diffuse into the material forced by the electric field during the interaction. iii) The *I*–*V* characteristics can be affected by oxidation and perturbations with a height above 1 µm can be easily formed by oxidation of a highly O-mobile material such as BiO*_x_*.

## Supporting Information

File 1 Scheme of electric circuit.
