# Supplementary material for: Kelvin probe force microscopy of the nanoscale electrical surface potential barrier of metal/semiconductor interfaces in ambient atmosphere
Source: Beilstein J Nanotechnol. 2019 Jul 15;10:1401–11. doi: 10.3762/bjnano.10.138 (PMC6664417; doi:10.3762/bjnano.10.138)
Supplement: File 1 — Scheme of electric circuit. [file Beilstein_J_Nanotechnol-10-1401-s001.pdf]

BEILSTEIN JOURNAL OF NANOTECHNOLOGY

Supporting Information

for

Kelvin probe force microscopy of the nanoscale electrical surface potential barrier of metal/semiconductor interfaces in ambient atmosphere

Petr Knotek, Tomáš Plecháček, Jan Smolík, Petr Kutálek, Filip Dvořák, Milan Vlček, Jiří Navrátil and Čestmír Drašar

Beilstein J. Nanotechnol. **2019,** *10,* 1401–1411. doi:10.3762/bjnano.10.138

Scheme of electric circuit

License and Terms: This is a supporting information file under the terms of the Creative Commons Attribution License (http://creativecommons.org/licenses/by/4.0). Please note that the reuse, redistribution and reproduction in particular requires that the authors and source are credited.
The license is subject to the *Beilstein Journal of Nanotechnology* terms and conditions: (https://www.beilstein-journals.org/bjnano)

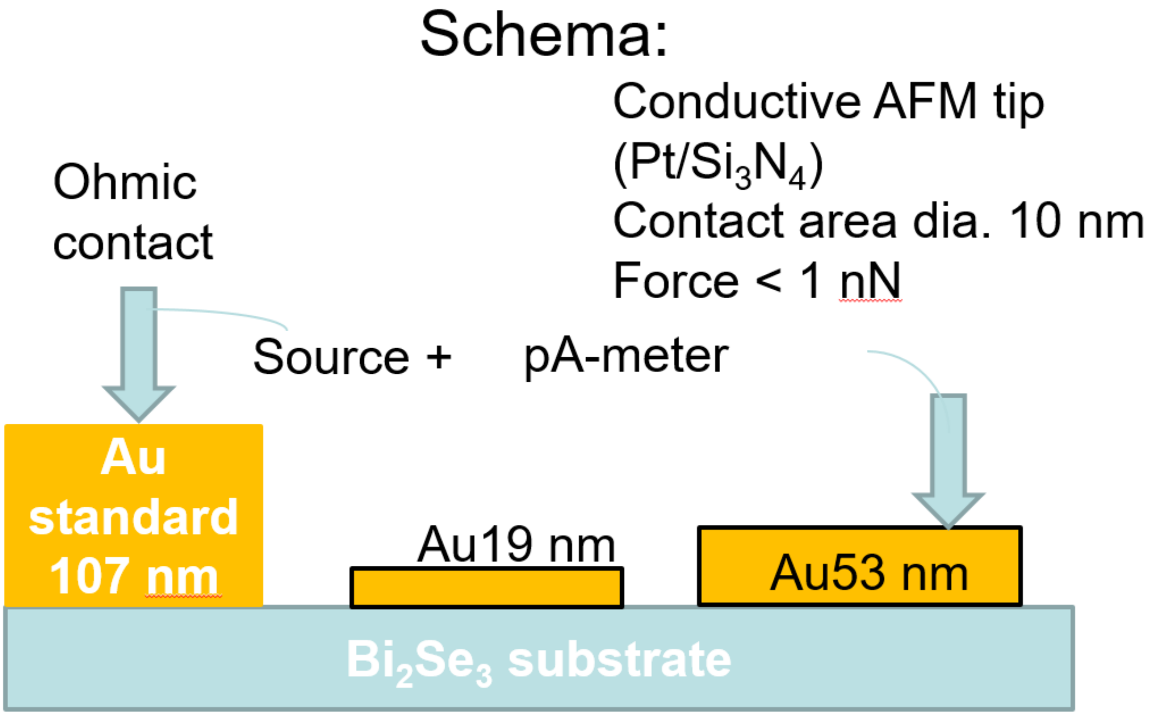

Figure S1: Scheme of electric circuit.
